# Changing power narratives: an exemplar case study on the professionalisation of community health workers in Liberia

**DOI:** 10.1136/bmjgh-2024-016351

**Published:** 2024-12-18

**Authors:** Anne Neumann, Marion Subah, Helene-Mari van der Westhuizen

**Affiliations:** 1 Nuffield Department of Primary Care Health Sciences, University of Oxford, Oxford, UK; 2 Charite Medical Faculty Berlin, Berlin, Berlin, Germany; 3 Ärzte für Madagaskar e.V, Dresden, Germany; 4 Liberia Country Office, Last Mile Health, Monrovia, Liberia; 5 Nuffield Department of Primary Care Health Sciences, Oxford University, Oxford, UK; 6 Centre for Tropical Medicine and Global Health, Medical Sciences Division, Oxford University, Oxford, UK; 7 TB Proof, Cape Town, South Africa

**Keywords:** Liberia, Health policy, Global Health, Health systems

## Abstract

Despite their central role in achieving health equity and Universal Health Coverage, only a minority of community health workers (CHWs) is formally recognised as health workforce and receives a salary. Community health policies are formed within the power dynamics of global health practice. We argue that critical investigations of the power dynamics that influence the design of CHW programmes can contribute system-level insights to strengthen their roles.

We present a national-level case study of the Liberian Community Health Assistant programme as an exemplar case of successfully introducing a nationwide CHW policy that professionalises CHWs. Using a theory of how power is exercised (Steven Lukes) for our analysis, we argue that Liberia’s success in overcoming external funder push-back on the payment of CHWs was enabled by strong political commitment and (re-)claiming government authority in and outside of decision-making processes. Consensus-building across government departments strengthened the government’s decision-making power. The availability and strategic use of suitable and contextualised evidence focused on the rights of CHWs allowed for proactive engagement with external funders’ concerns. To draw on learnings from the experience of Liberia, we recommend looking beyond the common effectiveness-oriented narratives in academic literature that focus on CHW’s functional role. By focussing on how power is exerted through policy negotiations around professionalisation, it could be possible to reframe conventional approaches to the role of CHW in other contexts as well.

**Summary box**

- Liberia has negotiated a national community health worker (CHW) programme that professionalises CHWs despite facing a fragmented national funding landscape.
- This country-level case study highlights the importance of aligning government champions across departments so that the government’s power in decision-making processes is amplified.
- The strategic use of evidence from the Liberian context, and critical questioning of existing ideological narratives was further key to advancing the development of this policy in the Liberian context.
- By using a theory of power to understand how the negotiations for this policy came about, we argue that it is important to reflect on what types of evidence are valued and who is invited to participate in the policy development process.

## Introduction

 Community health workers (CHWs) connect communities with the healthcare system. They contribute to equitable healthcare systems and universal health coverage by bridging geographic and cultural gaps in health service provision.^1^,^3^ CHW programmes were established to work where health services were not available, either due to health workforce shortages or the unequal distribution of health workforces.^1^ The idea of community involvement was enshrined in global health policy at the Alma-Ata Declaration in 1978, but its realisation has been heavily criticised.^14^,^6^

In political narratives, the role of CHWs is described as crucial for equitable and safe healthcare and as central to Universal Health Coverage and emergency responses to disease outbreaks.^6^,^10^ However, most CHWs around the world are employed in insecure and exploitative jobs and in many instances are expected to work as volunteers.^11^ Only about a sixth of CHWs receive a salary across 24 African countries, and continuous training and equipment are lacking.^7 12^ Recently, calls for professionalising CHWs to bridge this gap between political narratives and practice as well as to ensure CHWs’ access to human rights have become more prominent.^see e.g.^^313^,^15^ Professionalisation of the community health workforce could contribute towards more just and fair working conditions, including salaries and formal working agreements.^7^ Such an approach is supported by the current WHO guideline on CHW programmes^7^ and advocated for by CHWs themselves.^3^ This represents a shift from viewing CHWs not solely from a functional health agenda lens, but also from a labour rights and economic development perspective.

Liberia established a nationwide programme in 2016 to professionalise CHWs, which had to overcome many of the challenges that other countries with similar aspirations face.^16 17^ At national level, this exemplar case is an exception to the norm and demonstrates that a specific intervention is possible (a ‘positive deviant case’ in case study research).^18 19^ To investigate the political development of Liberia’s Community Health Assistant (CHA) programme, we draw from a publicly available report by Exemplars in Global Health, a policy-oriented research coalition^16^ and one of the authors’ (MS) first-hand experience in the negotiations of Liberia’s programme as the Country Director of the non-governmental organisation (NGO) Last Mile Health. We complement this with a document review, which helped to situate the case in the wider policy and research context and allowed to collate findings (see online supplemental appendix A).^20^ AN and HvdW came to this study with experience in CHW programmes in South Africa and Madagascar, which was relevant in establishing the transferability of findings.

Power imbalances play a major role in persisting injustice in global health and contribute to the dynamics of national policy negotiations.^1 21 22^ A recent study found that inequity shapes international cooperation between the Liberian government and donors (whom we will refer to as external funders).^23^ Several authors have recommended a greater focus on analysing power in global health and how it shapes national policy agendas in general and regarding CHW programmes in specific.^22 24 25^ In this analysis, we aim to understand the Liberian case of nationally professionalising CHWs using a theory of power—Lukes’ three faces of power^26^—and highlight insights that would be valuable for other settings.

## Introducing the community health worker programme in Liberia

Liberia’s 15 years of civil war until 2003 majorly disrupted the country’s education systems and state infrastructure including for health and contributed to a shortage of healthcare workers due to emigration.^16^ National policy has since focused on peacebuilding, increasing governance capacities, developing new infrastructure and stimulating economic growth. There was also strong interest in reactivating CHW programmes to fill health system gaps. However, these were implemented inconsistently by different stakeholders including NGOs, external funders and the government.^16^

The Ebola epidemic between 2014 and 2016 highlighted CHWs’ crucial role: they translated epidemic control measures to communities and reached areas that the health system would otherwise not be able to serve.^27^ The death toll among professionalised healthcare workers was high, which additionally put CHWs in key responder positions.^16^ Simultaneously, overall external funding peaked during this period of crisis.^16^

A pilot of a professional CHW cadre was conducted in Konobo district by the Liberian-based NGO Last Mile Health in 2012 and proved effective.^28^ The pilot’s data became the basis for redesigning Liberia’s community health policy in May 2015,^16^ for which all stakeholders working in community health were gathered.

**Figure 1:**
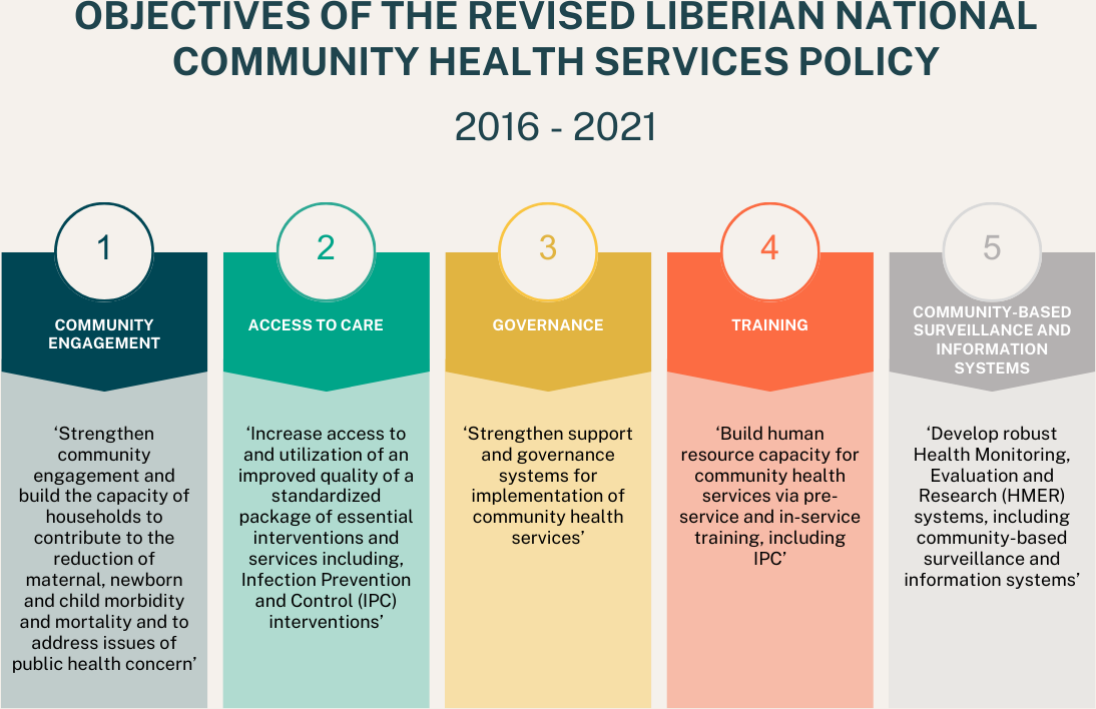
Objectives of the Revised National Community Health Services Policy 2016–2021.^29^

**Figure 2:**
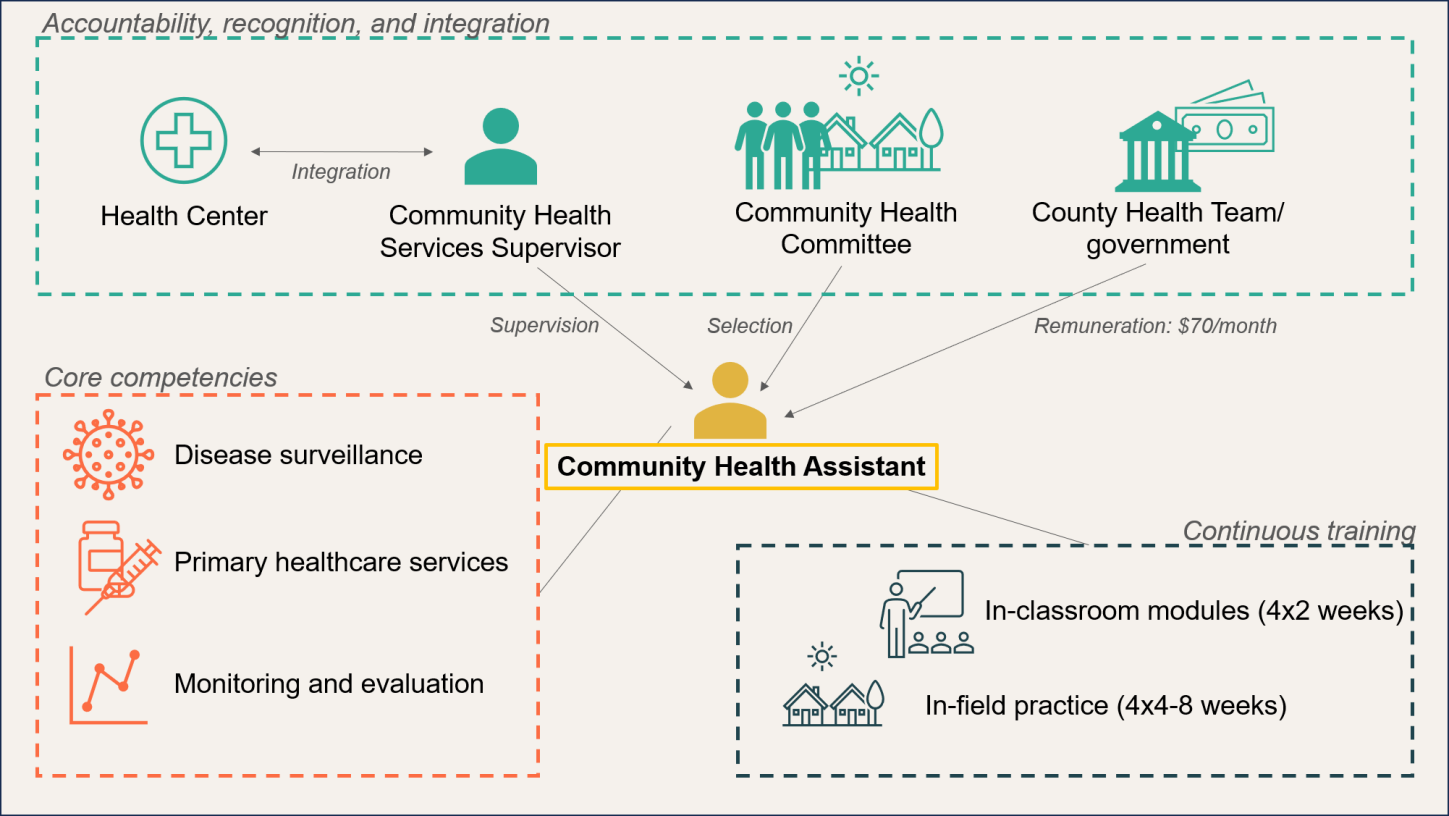
Key components of the Community Health Assistant programme according to the Revised National Community Health Services Policy 2016–2021 developed from Exemplars in Global Health’s investigation. *Note:* illustration of key components of the Liberian Community Health Assistant programme that include a clear definition of responsibilities, structured training and cross-linking with communities and the wider health system.^16^

The national CHW policy had five objectives around the improvement of health within the population, capacity-building and governance systems, and monitoring and evaluation (see figure 1).^29^ It established a nationwide, trained and paid CHW cadre called CHAs including structured and continuous training, a clearly defined scope of work and supervision. They were further formally integrated into the healthcare system.^16^ CHAs are selected by communities and serve areas that are more than five kilometres from a healthcare facility.^16 29^ NGOs, as ‘Ministry of Health (MoH) contractors’, pay professional midlevel healthcare providers (nurses, midwives and physician assistants) according to the MoH pay scale to work in health facilities as community health services supervisors, and CHAs receive a fixed monthly remuneration of $70 for 20 hours of work per week. However, CHAs are not currently recognised as civil servants, as originally advocated for by Last Mile Health representatives and the government. The programme is illustrated in figure 2.^16^

The first National Community Health Services Policy was commissioned until 2021 and recently updated for another 10 years (2023–2032).^30^ With the new policy, two additional cadres of CHWs will be professionalised.^30^ CHA cadres were extended to communities closer to health facilities and (peri-)urban communities.^30^ The accompanying strategy reinforces Liberia’s view of CHWs as central to its health system.^31^

## Understanding power and its three dimensions

Theories can help sharpen analyses and clarify the phenomenon under study. Lukes provides a theory of power that entails overt and subtle exercises of power and suits our investigation of actors in a policymaking process. Lukes expanded a two-dimensional view of power, which describes power over someone and power to set an agenda.^26 32^ He argued that there must be a third dimension of power, since not all forms of power lead to openly visible conflicts (see table 1).^26^ His third dimension of power denotes the power to make people *not* realise that they are being treated unjustly—it prevents people from seeing that someone or an organisation exerts power.^26^ While we deem Lukes’ theory of power a useful and accessible tool for this analysis, it must be noted that other theories may shed light on important other power issues such as more diffuse and structural exercises of power.^33^

**Table 1:** Lukes’ three dimensions of power

Dimension	Description of power mechanism
Decision-making power	Power executed through political decision-making from a position that enables A to make B do something against B’s interest and resulting in B’s dependency on A.
Non-decision-making power	Power to decide what is being discussed and decided in the first dimension; power to set the agenda.
Ideological power	Power at play through implicit values that lead to the normalisation of domination; power that makes others not complain about something they would complain about if being aware.

Explanations for each dimension of power as described by Lukes.^26^

## Understanding mechanisms: three dimensions of power in Liberia’s community health assistant programme development

The three dimensions of power help us understand the establishment of Liberia’s CHA policy as an exemplar case of a national, salaried and rights-based CHW programme. Key actors involved in setting up these policies were consortia led by the MoH, other ministries and CHWs (2023–2032 policy only).^16 31^ Additionally, external funders (including United States Agency for International Development, the Global Fund, the World Bank, humanitarian and anonymous funders) influenced the design as well as the NGO Last Mile Health.^16^

### Decision-making power: asserting the mandate of the Liberian government

The government by democratic mandate was in the position to execute this first dimension of power. The CHA policies set requirements for external funders’ in-country work, which earmarked funding for CHA salaries and allowed the pooling of funds to facilitate a nationally coordinated programme.^29 30^ These requirements were articulated through government-led negotiations, sparked by advocacy work of Last Mile Health. Negotiations within the government, led by the MoH, established a joint position between different government departments and ensured national political support. This position was championed by then-president Ellen Johnson Sirleaf on international platforms such as the US Senate Foreign Relations Committee.^16^

Throughout the negotiation process, the government issued public statements, which further signalled that ‘(the payment of CHWs) was not up for negotiation’.^16(p7)^ While this faced criticism from other global health actors (including external funders) for being too ambitious, the commitment of key political decision-makers across government departments was essential to implement this policy, especially when officeholders changed during this process.

### Non-decision-making power: negotiating the influence of external funding organisations

External funders, through their economic contributions to CHW programmes, were positioned to influence the discussion agenda. During negotiations, there was pushback from external funders against fixed remuneration and a civil servant status for CHWs, as they did not want to increase government spending on payrolls (30% of the budget at that time).^16^ Concerns about the programme’s sustainability were raised.

The Liberian government, however, asserted its agenda-setting power. *Exemplars in Global Health’*s investigation found that ‘Liberia proactively managed partner expectations and contingencies, appealed for donor transparency, and consistently pushed for its own vision and strategy. In the end, the country secured long-term financing for a program (sic) of its design’.^16(VI)^ The government’s argument was based on two premises drawing on experience from the Ebola epidemic: it had shown the importance of employment status to avoid demotivation and attrition, and it highlighted how CHWs are exposed to occupational risks like all healthcare workers, which underlies a moral argument for remuneration.^16^

Key to the government’s use of non-decision-making power were advocacy tools: first, the pilot conducted by Last Mile Health in 2012 with a similar design to the proposed approach.^16^ It provided the MoH with evidence of feasibility and advantages over other volunteer-based designs, with proof of scientific rigour through a peer-reviewed publication.^16 28^ A second tool was detailed financial planning. The MoH used granular data to inform funding negotiations.^16^ The investment case emphasised the positive effect on the labour market: the policy would lead to the employment of 4000 people who were mostly unemployed youth and women.^16^ This contributed to arguing for the policy’s positive and sustainable economic impact and proved to be a pivotable piece of evidence for the negotiation.

The Liberian government thus effectively and proactively managed the—quoting its own words—‘challenges around the alignment of differing agendas and priorities of the diverse stakeholders’.^31(p3)^ By using convincing financial and labour market arguments supplemented with real-world evidence, the government reached an agreement: CHWs would receive a salary of $70 but would not become civil servants to unburden the governments’ payroll commitments.

The 2023 policy update also incorporated experiences of CHW programmes during the COVID-19 pandemic, which was also reflected in the WHO guideline^7^ and changes in external funders’ stances towards CHW programmes.^34^ The priority of professionalised CHWs continued to be championed by the Liberian political leadership.

### Ideological power: what is considered evidence for policymaking?

Ideological power is less overt than the first two power dimensions. In this section, we consider what evidence is valued by whom. The Liberian government drew on locally generated evidence when developing its policy. This focus on evidence-based policymaking likely appealed to the external funder community, but also raises questions about what types of evidence were available and valued and what was dismissed. The academic literature on remunerating CHW draws on diverse ideological viewpoints. For example, some work is based on the assumption that CHW programmes should be viewed as health interventions whose (cost-)effectiveness ought to be maximised.^35^,^37^ Others draw on human rights-based arguments, placing CHWs as individuals with rights to secure employment and protection at work in the centre.^3 15 38 39^ Table 2 includes exemplary quotes depicting different narratives of CHWs in the academic literature. It is important to understand the implications of these perspectives and to be explicit about underlying values for evidence to be used appropriately in policy discussions. Further, unequal access to academic processes and resources (funding, publishing, etc) contributes to persisting injustices in who is perceived as credible and which analytical tools are available to explain observations.^40^ Evidence is context-sensitive and needs to be applied with careful consideration of the different policy environments.^41^

**Table 2:** Exemplary quotes for different narratives of community health workers in published academic literature

Premise	Exemplary quotes from academic literature
Effectiveness and efficiency-based narratives	‘There is evidence in the literature that incentives that are linked primarily to altruistic motivations lead to high rates of attrition over the long term, although there are certainly exceptions, as with the FCHVs in Nepal. This does not mean, however, that all CHWs must be made into salaried employees and have their financial needs fully met to sustain their engagement’.^35^ ‘Even stipends that are below the minimum or average wage in a community are often meaningful enough to keep CHWs, who might otherwise be completely unemployed, engaged in this work. Whether or not these stipends can be justified ethically or whether they are legal with respect to local labour law is a separate but important concern’.^35^ ‘A mix of financial and non-financial incentives, predictable for the CHWs, was found to be an effective strategy to enhance performance, especially of those CHWs with multiple tasks’.^37^
Rights-based narratives	‘Demanding that individuals volunteer in order to access healthcare for themselves, their family, and their community is an act of coercion.(…)The issue of whether to pay community health workers can no longer be framed as a policy choice about which reasonable minds can disagree. Community health workers from south Asia to southern Africa have long demanded fair compensation; it is well past time to cease giving those blocking their efforts technocratic cover’.^3^ ‘While labour rights have the potential to advance the health and well-being of CHWs, their absence remains a central barrier to the retention of this essential workforce and the achievement of universal health coverage. The lack of support, training opportunities, and adequate payment reduces the impact that health workers have on health outcomes, resulting in a weak health workforce and the undermining of universal health coverage’.^15^ ‘While increasingly the onus is on CHWs and CHW programmes to solve the problem of health access, attention should be given to the experiences of CHWs themselves. CHW programmes need to move beyond an instrumentalist approach to CHWs, and take a developmental and empowerment perspective when engaging with CHWs’.^50^ ‘As human resources, CHWs often become decontextualized objects of study and technical problems amenable to Western psychological, economic and management theories’^25^

The Liberian government engaged with evidence challenges by using in-country evidence based on the pilot led by Last Mile Health that already included rights-based elements, like recognition as healthcare workers and payment.^28^ It drew on cost-effectiveness arguments but intertwined these with justice arguments and thereby effectively defused narratives around volunteerism and heroism that have found legitimacy in many contexts.^42^,^44^

The perspectives of CHWs, and whether these are valued in the policymaking process, can perpetuate or challenge historically driven forms of ideological power. With little formal education, no access to labour rights and often coming from socioeconomically vulnerable contexts, many CHWs work within systems that inhibit them from developing critical perspectives on CHW programmes.^15^ In response, there has been a global trend of unionising among CHWs.^14^ CHWs themselves have challenged the established narratives around their roles and scope of work and have argued for the professionalisation of CHWs in political and academic spaces.^345^,^47^ Notably, CHWs were not included in the policy negotiations in 2015 but in 2023.^30 31^ This suggests that ideological power that underpins whose input is valued has also shifted through the course of the implementation of this policy.

## Learning from Liberia: considering power in other contexts

With the majority of CHWs globally working on a volunteer basis,^12^ the question of what other countries can learn from the Liberian government’s success in negotiating a national and professionalising CHW policy is highly relevant. Positive deviant (‘exemplar’) case studies do not provide *best practices* similar to a blueprint that will work in all contexts.^19^ However, they can be powerful foundations for advancing general knowledge beyond the case itself.^18^ The Liberian case study highlights the feasibility of professionalising CHWs amidst a programme that is largely externally funded. The alignment of government champions, the strategic development and use of local evidence, and the careful consideration of who shapes narratives (including academics and CHWs themselves) are key insights of this case study. Table 3 provides questions for reflection that could support the transferability of insights from our case study to other contexts facing similar challenges.

**Table 3:** Questions to critically assess the transferability of Liberia’s experience with designing the National Community Health Policy 2015-2021

Dimension of power	Suggested questions
Decision-making power	Who is in the position to promulgate a policy? What position does the government have towards professional CHW programmes? Who are political champions and influential partners that determine national and international negotiations? How does the government’s and its ministries’ political agenda affect CHW programmes? To what extent are positions on CHWs aligned between different government departments?
Non-decision-making power	Who influences what is being discussed as part of the policy? How do the multinational and international actors execute their influence? How influential are financial resources in negotiations? What evidence (context-derived, academic literature) is available? What and where are instrumentalist narratives? What role do rights-based narratives play?
Ideological power	How does the available evidence frame CHWs? Who has established the evidence? Local or international? Are there groups of CHWs (unions/advocacy groups) that could contribute to negotiations? Are stakeholders willing to include them?

CHW, community health worker.

Important context to consider is that the Ebola epidemic contributed to the prioritisation of the CHA programme in Liberia.^17^ The design phase had a beneficial timing since the Ebola pandemic led to increased external funding while illustrating both the importance and risk exposure of CHWs.^16^ Globally, the COVID-19 pandemic again led to increased interest in community health. We are encouraged by broader global shifts towards supporting this goal, including investment cases^48^ as well as the Monrovia Call to Action (2023)^14^ and the Community Health Delivery Partnership (2023), which express multiple stakeholders’ commitment to the professionalisation of CHWs.^49^

A limitation of our analysis is that we only focused on the design phase of the policy and not its implementation, which has its own power dynamics. Based on preliminary insights from one of the authors (MS), several aspects of the policy remain difficult to implement and to align throughout the country. Economic independence, and therewith sustainability remains a major point of discussion.^17^ While the use of the term ‘sustainability’ is being criticised by global health scholars,^1^ considerations of nations’ political economy will remain important in the establishment of long-term community health programmes. Other theories of power that focus on agency and structural exercises of power could further deepen the analysis of this case.

## Conclusion

The Liberian government, in collaboration with NGOs, successfully negotiated a national CHW programme that professionalised their role. Despite a fragmented CHW programme landscape and contradicting opinions on CHW salaries, Liberia succeeded in establishing a professional, national and primarily externally funded CHW programme within one year. Using Lukes’ three dimensions of power, we presented Liberia as a positive deviant case study of how different actors’ execution of power contributed to the design of the programme.

Liberia faced challenges that are likely to also occur in other contexts. Aligning standpoints between governmental departments, strategically producing and deploying evidence, and carefully considering how different types of evidence and perspectives are valued were important. Learning from these experiences can be useful for moving towards CHW programmes that shift beyond a functional perspective on CHWs and include rights-based perspectives, a focus on justice and the perspectives of CHWs themselves. Recent developments in academic literature, on the global health policy stage, and within CHW communities suggest that there may be further shifts in power dimensions that generate momentum to further advance the CHW professional role.

## Supplementary material

10.1136/bmjgh-2024-016351 online supplemental file 1

## Data Availability

Data are available upon request.
