# Supplementary material for: Changing power narratives: an exemplar case study on the professionalisation of community health workers in Liberia
Source: BMJ Glob Health. 2024 Dec 18;9(12):e016351. doi: 10.1136/bmjgh-2024-016351 (PMC11667280; doi:10.1136/bmjgh-2024-016351)
Supplement: online supplemental file 1 [file bmjgh-9-12-s001.pdf]

Appendix A

Documents included in the analysis

We added documents to our analysis of the case to put this case study in a wider context and contrast and verify findings from the core Exemplars in Global Health case study and MS's expertise against relevant documents. We found relevant documents based on topic expertise, references from other experts, and searching published literature and media outlets. Expanding our sources for analysis was particularly important for investigating Lukes' third dimension of power – ideological power.

**Policy documents**	**Relevance**
Ministry of Health. National Community Health Services Policy 2016-2021. Monrovia, Liberia, 2015.	Liberian policy context
Ministry of Health. National Community Health Program Policy 2023-2032. Monrovia, Liberia, 2023.	Liberian policy context
Ministry of Health. National Community Health Program Strategy 2023-2027. Monrovia, Liberia, 2023.	Liberian policy context
The Monrovia Call to Action. 2023. [Available from: https://chwsymposiumliberia2023.org/the-monrovia-call-to-action/(accessed].	Global policy context<br>Advocacy<br>Professionalisation of CHWs
WHO guideline on health policy and system support to optimize community health worker programmes. Geneva: World Health Organization, 2018.	Global policy context
**Case study on community health workers in Liberia**	
Chen N, Dahn B, Castañeda CL, et al. Community Health Workers in Liberia 2020 [Available from: https://www.exemplars.health/topics/community-health-workers/liberia accessed May 10 2023].	Core case study
**Academic literature (original research, academic books, and academic opinion pieces)**	
Ballard M, Odera M, Bhatt S, et al. Payment of community health workers. *The Lancet Global Health* 2022;10(9):e1242. doi: 10.1016/S2214-109X(22)00311-4	CHWs and rights<br>Volunteerism
Bhatia K. Community health worker programs in India: a rights-based review. *Perspectives in Public Health* 2014;134(5):276-82. doi: 10.1177/1757913914543446	CHWs and rights
Cakouros BE, Gum J, Levine DL, et al. Exploring equity in global health collaborations: a qualitative study of donor and recipient power dynamics in Liberia. *BMJ Global Health* 2024;9(3):e014399. doi: 10.1136/bmjgh-2023-014399	Liberian policy context

Closser S. Pakistan's lady health worker labor movement and the moral economy of heroism. *Annals of Anthropological Practice* 2015;39(1):16-28. doi: 10.1111/napa.12061	CHWs and rights<br>Volunteerism<br>Effectiveness narratives
Colvin CJ, Hodgins S, Perry HB. Community health workers at the dawn of a new era: 8. Incentives and remuneration. *Health Research Policy and Systems* 2021;19:1-25. doi: 10.1186/s12961-02100750-w	Effectiveness narratives
Healey J, Wiah SO, Horace JM, et al. Liberia's community health assistant program: scale, quality, and resilience. *Global Health: Science and Practice* 2021;9(Supplement 1):S18-S24. doi: 10.9745/GHSP-D-20-00509	Liberian policy context
Kane S, Kok M, Ormel H, et al. Limits and opportunities to community health worker empowerment: a multi-country comparative study. *Social Science & Medicine* 2016;164:27-34. doi: 10.1016/j.socscimed.2016.07.019	CHWs and rights
Kok MC, Dieleman M, Taegtmeyer M, et al. Which intervention design factors influence performance of community health workers in low-and middle-income countries? A systematic review. *Health Policy and Planning* 2015;30(9):1207-27. doi: 10.1093/heapol/czu126	Effectiveness narratives
Luckow PW, Kenny A, White E, et al. Implementation research on community health workers' provision of maternal and child health services in rural Liberia. *Bulletin of the World Health Organization* 2017;95(2):113. doi: 10.2471/BLT.16.175513	Liberian policy context
Maes K. Community health workers and social change: An introduction. *Annals of Anthropological Practice* 2015;39(1):1-15. doi: 10.1111/napa.12060	History of CHWs<br>CHWs and rights
Maes K. The lives of community health workers: Local labor and global health in urban Ethiopia: Routledge 2016.	CHWs and rights
Mukherjee J. An introduction to global health delivery: practice, equity, human rights: Oxford University Press New York 2021.	History of CHWs<br>CHWs and rights
Prince RJ, Prince R, Brown H. Volunteer economies: The politics & ethics of voluntary labour in Africa: Boydell & Brewer 2016.	Volunteerism
Shanthosh J, Durbach A, Joshi R. Charting the Rights of Community Health Workers in India: The Next Frontier of Universal Health Coverage. *Health and Human Rights* 2021;23(2):225.	CHWs and rights
Siekmans K, Sohani S, Boima T, et al. Community-based health care is an essential component of a resilient health system: evidence from Ebola outbreak in Liberia. *BMC Public Health* 2017;17:1-10. doi: 10.1186/s12889-016-4012-y	Liberian policy context
Trafford Z, Swartz A, Colvin CJ. "Contract to volunteer": South African community health worker mobilization for better labor protection. *New Solutions: A Journal of Environmental and Occupational Health Policy* 2018;27(4):648-66. doi: 10.1177/1048291117739529	Volunteerism<br>CHWs and rights

Wennerstrom A, Smith DO. Labour exploitation among community health workers. *The Lancet Global Health* 2023;11(10):e1484-e85. doi: 10.1016/S2214-109X(23)00409-6	CHWs and rights<br>Advocacy
**Reports and media coverage**	
A tireless advocate for integrating community health workers into Kenya's primary care system: Johnson & Johnson Center for Health Worker Innovation; 2023 [Available from: https://chwi.jnj.com/news-insights/a-tireless-advocate-for-integrating-community-health-workers-into-kenyas-primary-care-system accessed 8 May 2024].	Advocacy<br>CHWs and rights<br>Volunteerism
Bazira L, Muyingo P. Compensating community-health workers: Project Syndicate; 2023 [Available from: https://www.project-syndicate.org/commentary/women-community-health-workers-must-be-paid-by-lennie-bazira-and-prossy-muyingo-2023-10?barrier=accesspaylog accessed May 8 2024].	Advocacy<br>CHWs and rights<br>Volunteerism
Colvin D. What motivates community health workers? Designing programs that incentivize community health worker performance and retention. *Developing and strengthening community health worker programs at scale: a reference guide for program managers and policy makers Washington, DC: USAID/Maternal and Child Health Integrated Project (MCHIP)* 2014.	Effectiveness narratives
Community health workers unite for recognition, rights, and respect Public Services International2023 [Available from: https://publicservices.international/resources/news/community-health-workers-unite-for-recognition-rights-and-respect?id=14032&lang=en accessed 8 May 2024].	Advocacy<br>CHWs and rights
Dahn B, Woldemariam AT, Perry H, et al. Strengthening primary health care through community health workers: Investment case and financing recommendations. 2015. [Available from: https://chwcentral.org/wp-content/uploads/2015/09/CHW-Financing-FINAL-July-15-2015.pdf(accessed].	Global policy context
Nepomnyashchiy L, Westgate C, Wang A, et al. Protecting community health workers: PPE needs and recommendations for policy action. *Center for Global Development* 2020; 15. https://www.cgdev.org/publication/protecting-community-health-workers-ppe-needs-and-recommendations-policy-action (accessed 8 May 2024).	CHWs and rights<br>Advocacy
About the CDHP: Community Health Delivery Partnership; n.d. [Available from: https://www.communityhealthdeliverypartnership.org/about-chdp accessed 14 Oct 2024	Global policy context
Sirleaf EJ, Clark H. Report of the Independent Panel for Pandemic Preparedness and Response: making COVID-19 the last pandemic. *The Lancet* 2021;398(10295):101-03. doi: 10.1016/S0140-6736(21)01095-3	Global policy context

The Global Fund. Key Performance Indicators (KPIs): Handbook for the 2023-2028 Strategy. 2023. https://www.theglobalfund.org/media/12681/strategy_globalfund2023-2028-kpi_handbook_en.pdf.	Global policy context
